# Incidence, Phenotypic Features and Molecular Genetics of Kallmann Syndrome in Finland

**DOI:** 10.1186/1750-1172-6-41

**Published:** 2011-06-17

**Authors:** Eeva-Maria Laitinen, Kirsi Vaaralahti, Johanna Tommiska, Elina Eklund, Mari Tervaniemi, Leena Valanne, Taneli Raivio

**Affiliations:** 1 Children's Hospital, Helsinki University Central Hospital, University of Helsinki, FI-00029 Helsinki, Finland; 2 Institute of Biomedicine, Department of Physiology, Biomedicum Helsinki, University of Helsinki, FI-00014 Helsinki, Finland; 3 Helsinki Medical Imaging Center, Helsinki University Central Hospital, FI-00029 Helsinki, Finland

## Abstract

**Background:**

Kallmann syndrome (KS), comprised of congenital hypogonadotropic hypogonadism (HH) and anosmia, is a clinically and genetically heterogeneous disorder. Its exact incidence is currently unknown, and a mutation in one of the identified KS genes has only been found in ~30% of the patients.

**Methods:**

Herein, we investigated epidemiological, clinical, and genetic features of KS in Finland.

**Results:**

The minimal incidence estimate of KS in Finland was 1:48 000, with clear difference between males (1:30 000) and females (1:125 000) (*p *= 0.02). The reproductive phenotype of 30 probands (25 men; 5 women) ranged from severe HH to partial puberty. Comprehensive mutation analysis of all 7 known KS genes (*KAL1*, *FGFR1*, *FGF8*, *PROK2*, *PROKR2*, *CHD7*, and *WDR11*) in these 30 well-phenotyped probands revealed mutations in *KAL1 *(3 men) and *FGFR1 *(all 5 women vs. 4/25 men), but not in other genes.

**Conclusions:**

Our results suggest that Finnish KS men harbor mutations in gene(s) yet-to-be discovered with sex-dependent penetrance of the disease phenotype. In addition, some KS patients without *CHD7 *mutations display CHARGE-syndrome associated phenotypic features (e.g. ear or eye anomalies), possibly implying that, in addition to *CHD7*, there may be other genes associated with phenotypes ranging from KS to CHARGE.

## Introduction

Kallmann syndrome (KS; MIM# 147950), a combination of congenital hypogonadotropic hypogonadism (HH; MIM# 146110) and decreased/absent sense of smell, results from disturbed intrauterine migration of gonadotropin-releasing hormone (GnRH) neurons from the olfactory placode to the hypothalamus [1-3]. Patients with KS usually lack puberty, but the reproductive phenotype may vary from severe hypogonadism (cryptorchidism or micropenis in male infants) to reversal of hypogonadotropism later in life [4,5]. Associated phenotypic features include cleft lip/palate, hearing impairment, dental agenesis, limb anomalies, renal agenesis, and mirror movements [6]. The incidence estimates of KS are scarce and variable, and the condition appears to be 3-5 times more frequent in men [6-8].

KS is genetically heterogeneous, and the majority of cases (~60%) present as sporadic cases (only one person affected in the family). In familial KS, autosomal recessive, autosomal dominant, and X-chromosomal recessive inheritance have been described [9]. Oligogenic mode of inheritance has also been suggested [10-13]. The genes involved in the etiology of KS are *KAL1 *[14,15], *FGFR1 *[16], *FGF8 *[11], *PROK2 *[13,17], *PROKR2 *[13,17], and *WDR11 *[18]. A monoallelic mutation in *FGFR1 *underlies approximately 10% of KS cases [16]. Consistent with variable expressivity of *FGFR1 *mutations, the mutation carriers may also display spontaneous fertility [16,19-21], and loss-of-function mutations in *FGFR1 *are found in 7% of patients with congenital HH but reportedly normal sense of smell [5]. In addition, mutations in *CHD7, *underlying over 60% of CHARGE syndrome (coloboma, heart defects, choanal atresia, retarded growth and development, genital abnormalities, and ear anomalies; MIM# 214800) [22,23], have also shown to cause KS. To date, a molecular genetic diagnosis is attained in only approximately 30% of KS patients [24,25], which implies the existence of additional genes underlying KS.

Here we provide a nationwide minimal incidence estimate of KS in Finland, describe the phenotypic and molecular genetic features of a representative series of Finnish KS patients, and characterize the functional effects of novel *FGFR1 *missense mutations.

## Materials and methods

### Data sources

Discharge registers of all 5 university hospitals in Finland were queried by the International Classification of Disease (ICD) edition 10 and 9 (ICD-10, -9) codes for hypogonadotropic hypogonadism (E23.04 and 253.4, respectively) covering the years from 1996 to 2007. Among the query results, in any case of hypogonadotropic hypogonadism, the medical charts were manually revised, and all patients of Finnish origin who had been diagnosed with KS on the basis of isolated gonadotropin deficiency without an organic cause, and anosmia or hyposmia, were identified. Of note, anosmia or hyposmia had been diagnosed on the basis of anamnestic information, olfactometry, or by the inability to recognize familiar odors such as coffee. All except one (excluded from further calculations), were born between 1976 and 1987. Thus the final series of hereby identified KS patients reflects the number of KS patients born in Finland between 1976 and 1987. To estimate a national incidence of KS, this number of KS patients was compared to the number of live-born children in Finland between 1976 and 1987 (Statistics Finland register database at http://pxweb2.stat.fi/database/StatFin/vrm/synt/synt_en.asp).

### Clinical and molecular genetic evaluation of the patients

#### Medical history and clinical examination

The patients willing to participate in the molecular genetic part of this study were enrolled from the 5 different university hospitals in Finland, and were asked for a detailed medical history including history of cryptorchidism, micropenis, prior pubertal development, prior treatment, associated phenotypes, and the sense of smell, and these data were verified by medical chart review. Patients were also asked for the presence or absence of KS, normosmic HH, infertility, isolated anosmia or hyposmia, cryptorchidism, delayed puberty, dental agenesis, or cleft lip and palate in their family members and relatives. In retrospect, all adult patients fulfilled the following criteria: 1) absent or incomplete pubertal development by the age of 18 yrs, 2) low circulating basal sex steroid levels in association with inappropriately low or normal gonadotropin levels, and subnormal or normal response to GnRH stimulation test, 3) otherwise normal anterior pituitary function, 4) anosmia or hyposmia based on either anamnestic information, formal testing (e.g. olfactometry), or testing with familiar odors, and 5) no organic cause for their condition. In addition to adult patients, 12-18 yr-old-patients with unequivocal signs of severe congenital HH (history of cryptorchisim and/or micropenis), absent puberty, and anosmia/hyposmia were enrolled.

The participants underwent a complete physical examination, including mirror movement assessment, and measurement of testicular volume with a ruler (length × width^2 ^× 0.52). Olfaction was assessed with the 40-item smell testing (University of Pennsylvania Smell Identification Test, UPSIT, Sensonics Inc, Haddon Heights, NJ), and individuals scoring <5^th ^percentile for age in UPSIT, were classified as anosmic. A blood sample was drawn for DNA extraction. Renal structures were assessed by abdominal ultrasound scan. The following brain magnetic resonance imaging (MRI) protocol was used to visualize the olfactory bulbs, sulci, and inner ears (corresponding sequences in 1.5 and 3 tesla units): axial T2 FSE and FLAIR images of the whole brain, coronal T2 FSE with 3 mm slice thickness starting from the anterior surface of the frontal lobe, 3D MPR sagittal images (1 × 1 mm) covering the whole head with coronal reconstructions, 3D T2-weighted thin slices (CISS, DRIVE, voxel size 0.3-0.5 mm × 3) axial images from the region of the inner ear. No contrast medium was used.

The family members willing to participate were contacted and recruited with the permission of the proband, and they filled in a questionnaire or were interviewed by telephone for prior pubertal development, fertility, associated phenotypes, and the sense of smell. A blood sample was drawn for DNA extraction.

#### Mutation analysis

Genomic DNA from peripheral blood leukocytes was extracted, and the coding exons and exon-intron boundaries of 8 genes [*KAL1 *(MIM# 308700; RefSeq NM_000216.2, gi:119395745), *FGFR1*(MIM# 136350; RefSeq NM_023110.2, gi:105990521), *FGF8 *(MIM# 600483; RefSeq NM_033163.3, gi:298919216), *PROK2 *(MIM# 607002; RefSeq NM_001126128.1, gi:187167260), *PROKR2 *(MIM# 607123; RefSeq NM_144773.2, gi:30581162), *NELF *(MIM# 608137; RefSeq NM_001130969.1, gi:195972908) [26], *CHD7 *(MIM# 608892; RefSeq NM_017780.2, gi:54112402) and *WDR11 *(MIM# 606417; RefSeq NM_018117.11, gi:284172506)] were PCR-amplified and screened by direct sequencing. In *FGFR1, *both exons 8A and 8B, generating isoforms FGFR1-IIIb and FGFR1-IIIc by alternative splicing [27], respectively, were screened. *FGFR1 *and *CHD7 *were also analyzed by multiplex ligation-dependent probe amplification assay (MLPA). MLPA was performed according to manufacturer's protocol (Salsa MLPA Kits P133 Kallmann-2 and P201-B1 CHARGE, MRC-Holland, Amsterdam, the Netherlands). All primer sequences and PCR conditions are available upon request. Nonsense changes resulting in a truncated protein, nucleotide changes affecting splice sites, and frameshifting insertions or deletions were categorized as pathogenic mutations. Missense changes, which were absent from the Single Nucleotide Polymorphism database (http://www.ncbi.nlm.nih.gov/projects/SNP/) and from at least 100 controls from the same geographical region, were identified as possibly pathogenic mutations, and characterized further by functional *in vitro *studies. Mutations were confirmed from a second PCR product. Mutation nomenclature is according to the guidelines of the Human Genome Variation Society [28] and was verified using the Mutalyzer software (http://www.mutalyzer.nl/2.0/).

This study was performed with appropriate permissions from the Ethics Committee (E7) of the Helsinki University Central Hospital, and from each university hospital in Finland. Written informed consents were obtained from the participants, and also from their guardian if the participant was less than 16 yrs of age.

#### Site-directed mutagenesis

N-terminal myc-tagged FGFR1c cDNA in pcDNA3.1+ was used as a template for site-directed mutagenesis using QuikChange II XL site-directed mutagenesis kit (Stratagene, La Jolla, CA). Following mutagenesis, the sequence of the whole plasmid, including the presence of the mutation (G48S, R209H, or E670A), was verified. The myc-tagged FGFR1c cDNA was used for all *in vitro *studies, as described [5].

#### Transfections

All transfections were performed with 300 ng of DNA (50 ng of myc-tagged WT or mutated FGFR1 cDNA and 250 ng of empty vector (EV)) in 24-well plates, using FuGene HD transfection reagent (Roche Diagnostics GmbH, Mannheim, Germany) according to the manufacturer's instructions.

#### Receptor expression and maturation studies

For endoglycosidase digestions, sub-confluent COS-1 cells were transiently transfected. 24 h later, cells were washed with phosphate buffered saline (PBS) and lysed with 100 μl of radioimmunoprecipitation assay buffer (Sigma, Saint Louis, MO) containing 1X Halt protease inhibitor cocktail (Pierce, Rockford, IL). For deglycosylation analysis, 5 μg of protein (Protein Quantification kit-Rapid (Sigma-Aldrich Chemie GmbH, Buchs, Switzerland)) was subjected to PNGasef and EndoH_f _digestion according to manufacturer's recommendations (New England Biolabs, Ipswich, MA).

#### Western blot analysis

Both untreated or endoglycosidase-treated samples were resolved on PAGEr Gold Precast 4-20% Tris-Glycine Gels (Lonza, Rockland, ME), and subjected to western blot analysis using an anti-myc antibody (1:1000, clone 4A6, Millipore, Billerica, MA). Immunoreactivity was visualized using Amersham ECL Western blotting detection reagents (GE Healthcare Limited, Buckinghamshire, UK). To control for equal loading, blots were stripped (Restore Western Blot Stripping Buffer (Pierce, Rockford, IL)), and reprobed using anti-β-actin primary antibody (1:1000, Santa Cruz Biotechnology, Santa Cruz, CA). Overall expression levels were visualized from the PNGase-treated samples, and receptor maturation patterns from the EndoH_f_-treated samples. Endoglycosidase experiments were repeated at least three times.

#### Cell-surface expression

Cell-surface expression was determined with ELISA technique. Sub-confluent COS-1 cells were transiently transfected, and, 24 h later, washed with PBS, fixed with 4% paraformaldehyde in PBS for 15 min, and blocked with PBS, containing 1% bovine serum albumin, for 1 h. Cell-surface expression levels were determined using an anti-myc primary-antibody (see above), HRP-conjugated secondary antibody and assayed using 3,3`,5,5`tetramethylbenzidine (Sigma, Saint Louis, MO) as the substrate with detection at 450 nm following the addition of 0.5 M H_2_SO_4 _[29]. Experiments were performed in triplicate and repeated at least three times.

#### MAPK signaling studies

L6 myoblasts were transiently transfected at 10% confluency. 5 h later, culture medium was replaced with starvation media (2% fetal bovine serum). 20 h later, the cells were stimulated with FGF2 (Cell Signaling Technology, Danvers, MA), 50 ng/ml for 0/2/10/30 min. At the indicated time points, the cells were washed with ice-cold PBS and lysed with 50 μl of radioimmunoprecipitation assay buffer (Sigma, Saint Louis, MO), containing 1X Halt phosphatase inhibitor cocktail (Pierce, Rockford, IL), and lysates of 3 replicate wells at each time point were pooled for western blot analyses. Samples containing equal amounts of protein (8 μg) were resolved as described above, and subjected to western blot analysis using a phospho-p44/42 MAPK (Thr202/204) antibody (1:1000, Cell Signaling Technology, Danvers, MA). Immunoreactivity was visualized as described above. To control for equal loading, blots were reprobed using a p44/42 MAPK primary antibody (1:1000, Cell Signaling Technology, Danvers, MA). Experiments were repeated at least three times.

### Statistical analyses

Fisher's exact test was used to determine statistical significance (Simple Interactive Statistical Analysis, SISA, http://www.quantitativeskills.com/sisa/). All *p*-values are two-sided, and *p *< 0.05 was considered significant.

## Results

### Minimal estimate of the incidence of KS in Finland

Based on the hospital databases, between 1996 and 2007, altogether 16 KS patients (13 boys and 3 girls) of Finnish origin had been diagnosed in the 5 university Hospitals in Finland. They were born between 1976 and 1987, when altogether 767 778 infants were born alive in Finland. Thus, the minimal estimate of the overall incidence of KS was 16/767 778 (1 in 48 000 newborns). There was a clear difference in estimates between boys (13/392 900; 1 in 30 000) and girls (3/374 878; 1 in 125 000) (*p *= 0.02).

### Molecular genetic and clinical features of KS patients

Thirty probands (25 men and 5 women) participated in the molecular genetic part of our study (mean age 37 yrs, range 13-61 yrs). Two of the men were of other than Finnish origin. Of note, the male-to-female ratio among these participants was not different from that of the patients diagnosed between 1996 and 2007 (see above). Fifteen of the 30 probands (50%) had at least one relative with cleft lip and/or palate, isolated anosmia, normosmic HH, or KS. All Finnish KS subjects displayed unequivocal anosmia as tested with the 40-item UPSIT test. Overall, 12 of 30 (40%) probands carried a mutation in either *FGFR1 *(9 probands) or *KAL1 *(3 probands) (Table 1). No mutations were detected in *PROK2*, *PROKR2*, *FGF8 *or *WDR11*. Importantly, all 5 women had *FGFR1 *mutations, in contrast to only 4 of 25 men (Table 1) (*p *= 0.0009). This difference in the prevalence of *FGFR1 *mutation carriers among men and women affected by KS persisted statistically significant when analyses included only probands of Finnish origin (5 women, 3/23 men) (*p *= 0.0006), or those of Finnish origin without a *KAL1 *mutation (5 women, 3/20 men) (*p *= 0.001). No intragenic aberrations were found in MLPA analysis of *FGFR1*; all the *FGFR1 *mutations were detected by standard sequencing.

**Table 1:** Clinical and molecular genetic features of the probands with Kallmann Syndrome.

			**Mutation**			**History of**			**Olfaction**		
						
**Proband**	**Sex**	**Family history**	**Gene**	**Nucleotide change**	**Predicted effect**	**Micro- penis**	**Cryptor- chidism**	**Puberty**	**SIT**	**MRI of OB**	**Associated phenotypes in physical examination**
				
1	F	No	*FGFR1*	c.246_247delAG*	p.E84GfsX26			No^#^	A	Absent	A missing tooth
11	F	KS, nHH	*FGFR1*	c.142G > A	p.G48S			No	A	NA	None
45	F	Infertility	*FGFR1*	c.961_962delAA	p.K321RfsX13			No	A	Absent l.dx, small l.sin	None
503	F	KS	*FGFR1*	c.1305_1306dupAT	p.S436YfsX3			No	A	Absent	None
54	F	KS	*FGFR1*	c.1825C > T	p.R609X			No	A	Absent	Cleft lip, two missing teeth, scoliosis
13	M	No	*FGFR1*	c.626G > A	p.R209H	Yes	No	No	A	NA	None
14	M	KS	*FGFR1*	c.961_962delAA	p.K321RfsX13	NA	No	Partial^§^	A	Rudimentary	None
20	M	nHH^¤^	*FGFR1*	c.2009A > C	p.E670A	Yes	No	No	A	NA	Cleft lip and palate
50	M	KS, nHH	*FGFR1*	c.11G > A	p.W4X	NA	No	No	NA	NA	None
2	M	KS	*KAL1*	g.2357_2360delAgta		Yes	Bilat	No	A	NA	Synkinesia, a missing tooth
4	M	KS	*KAL1*	c.784C > T	p.R262X	Yes	Bilat	No	A	NA	Synkinesia, left renal agenesis, rheumatoid arthritis
9	M	KS	*KAL1*	c.471_472delCT	p.S158WfsX45	NA	R	No	A	NA	Synkinesia, unilateral conductive hearing loss

*de novo -mutation; ^#^Testicular volume less than 4 mL in males or no spontaneous thelarche in females; ^§^Testicular volume 6 mL at the age of 33; ^¤^A brother with normosmic HH, cleft lip and palate, and limb anomalies.

SIT, The Smell Identification Test; OB, olfactory bulbs; F, female; M, male; nHH, normosmic HH; R, right; A, anosmia; NA, not assessed

*FGFR1 *MIM# 136350; RefSeq NM_023110.2, gi:105990521

*KAL1 *MIM# 308700; RefSeq NM_000216.2, gi:119395745

**Figure 1:**
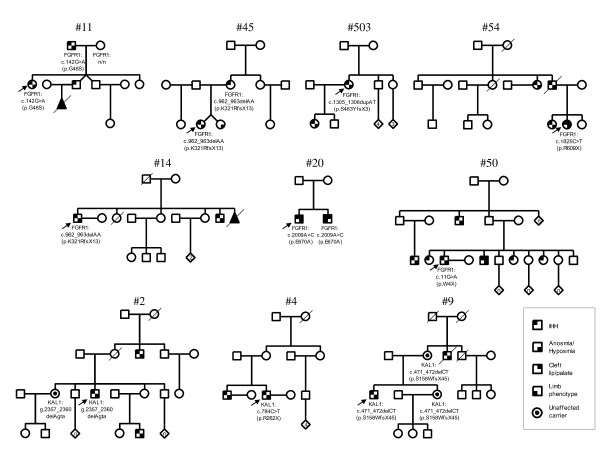
**Pedigrees of KS patients carrying an *FGFR1 *or *KAL1 *mutation**. Pedigrees #11, #503, #45, #54, and #50 are consistent with autosomal dominant form of inheritance. X-chromosomal recessive inheritance is apparent in pedigrees #2 and #9.

Variable expressivity of *FGFR1 *mutations was noticed within families (Figure 1). The proband #11 with a previously reported *FGFR1 *missense mutation (c.142G > A [p.G48S]) [20], had a severe congenital HH (see Table 1). However, her anosmic father carrying the same mutation had had four children during testosterone therapy, consistent with active spermatogenesis, and her brother had normosmic HH. Also, the associated phenotypes among *FGFR1 *mutation carriers varied. The proband #20, and his brother with normosmic HH, had the same missense mutation (c.2009A > C [p.E670A]); the brother also had cleft lip and palate, and limb malformations manifested as a fusion of 3^rd ^and 4^th ^metatarsal bones, and missing of the 2^nd ^and 3^rd ^toe in both feet (see Table 1). In addition, proband #503 with a frameshift mutation (c.1305_1306dupAT [p.S436YfsX3]) had two children following assisted reproductive techniques, and her daughter also had KS. Proband #1 was the only proven carrier of *de novo FGFR1 *mutation. The parents of probands #13, #14, #20, #50, #54, and #503 were unavailable for the mutation analysis.

Three probands had mutations in *KAL1 *(Table 1). In two pedigrees, the X-chromosomal recessive mode of inheritance was apparent (Figure 1). The sister of the proband #2, and the mother and the sister of the proband #9 were all unaffected heterozygous carriers of a *KAL1 *mutation.

One proband carried a novel missense change in *CHD7 *(c.7988C > T [p.A2663V), not present in 100 controls. This variant was predicted to be benign by PolyPhen [30] (http://genetics.bwh.harvard.edu/pph/), and it is therefore unlikely that this is a disease-causing mutation. *CHD7 *was also analyzed by MLPA because intragenic *CHD7 *deletions have been found in up to 22% of CHARGE patients [31,32], but none were detected in our KS patients.

In addition, novel missense changes in *NELF *(c.280 G > A [p.G94S], c.1514 C > T [p.T505M]) were found in probands carrying an *FGFR1 *mutation (proband #13: G94S, proband #54: T505M). However, these were also present in the controls (G94S: 6/100, T505M: 1/100).

### Functional characterization of mutant FGFR1s

To examine the functional consequences of the *FGFR1 *missense mutations G48S, R209H, and E670A, we compared total and cell-surface expression, receptor maturation, and signaling activities of WT and mutant receptors.

#### Overall expression and receptor maturation

**Figure 2:**
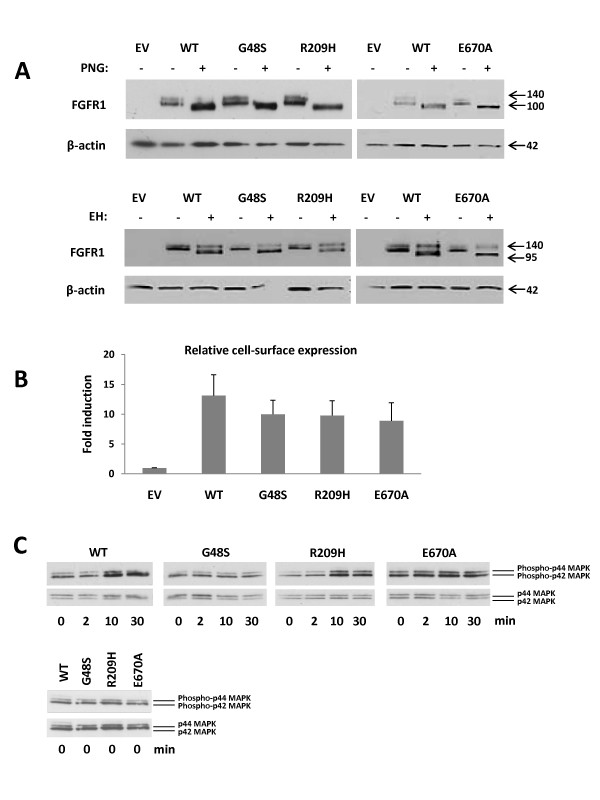
**Functional analyses of FGFR1 mutants**. **A**. Endoglycosidase analysis of mutant FGFR1s. COS-1 cells were transiently transfected with myc-tagged WT or mutated FGFR1 cDNA. EV= empty vector. Cell lysates were subjected to PNGase (PNG, upper panel) or EndoH_f _(EH, lower panel) digestion. The overall expression of the G48S, R209H and E670A was not significantly decreased as compared to WT (PNGase-treated bands). Receptor maturation patterns are shown in lower panel. The G48S, R209H and E670A mutants have a similar maturation pattern as WT receptor. **B**. Cell-surface expression of FGFR1 mutants. COS-1 cells were transiently transfected with myc-tagged WT or mutated FGFR1 cDNA. EV = empty vector. Cell-surface expression levels were determined from fixed cells using an anti-myc primary antibody. Absorbancies were detected at 450 nm. The values on the Y-axis represent fold inductions as compared to the level elicited by EV. The WT, G48S, R209H and E670A have similar cell-surface expression levels. **C**. MAPK signaling analysis of FGFR1 mutants. L6 myoblasts were transiently transfected with myc-tagged WT or mutated FGFR1 cDNA and treated with FGF2 for 0/2/10/30 min. Phospho-specific antibodies (phospho-p44/42 MAPK) were used to determine phosphorylation of MAPK. To control for equal loading, blots were reprobed using an anti p44/42 MAPK antibody. WT and R209H show clear phosphorylation of MAPK after 10 and 30 minutes of FGF2 treatment. With the mutant receptors G48S and E670A, no clear phosphorylation of MAPK was seen in any of the indicated time points. For comparison of baseline activities, all untreated samples (0 min) were also run on the same gel.

Western blot analysis showed two immunoreactive specific bands for wild-type (WT) FGFR1 at approximately 140 kDa and 120 kDa (Figure 2A). Removal of all types of N-linked carbohydrate chains with PNGase digestion reduced the bands into a single one of ~100 kDa. The overall expression of the mutant FGFR1s G48S, R209H, and E670A, as judged from the PNGase-treated samples, was not significantly decreased, as compared to WT (Figure 2A, upper panel). Endoglycosidase H (EndoH_f_) treatment, which removes only high-mannose N-linked sugars typical for immature forms of the receptor, resulted in similar maturation patterns for G48S, R209H, and E670A, as compared to WT, as only the minor 120 kDa bands changed mobility (Figure 2A, lower panel). This indicates that this minor band represents the partially processed receptor, whereas the 140 kDa EndoH_f _resistant band represents the fully glycosylated, mature form of FGFR1.

#### Cell-surface expression

Cell-surface expression of the WT receptor and the mutants G48S, R209H, and E670A were examined in COS-1 cells. Consistent with the results of deglycosylation experiments, the G48S, R209H, and E670A mutants had similar cell-surface expression levels as WT (Figure 2B).

#### MAPK signaling

The signaling activities of the FGFR1 mutants G48S, R209H and E670A were assayed in L6 myoblasts, a cell line largely devoid of endogenous FGFRs and FGFs [33]. Cells were transfected with WT or mutant FGFR1s, and treated with FGF2 for 0/2/10/30 min. Cells expressing WT receptor showed clear phosphorylation of MAPK after 10 and 30 min of FGF2 treatment (Figure 2C). No ligand-induced phosphorylation of MAPK was seen in cells transfected with G48S and E670A, whereas the R209H responded to FGF2 treatment similarly to WT (Figure 2C). All untreated samples were also run on the same gel, and did not display differences in MAPK phosphorylation, indicating similar baseline activities (Figure 2C).

### Features of KS patients without *KAL1* or *FGFR1* mutations

**Figure 3:**
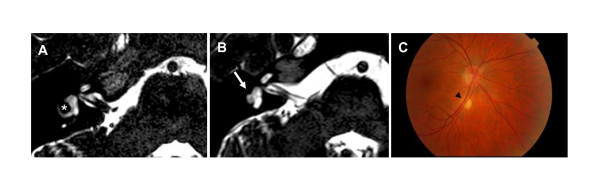
**The axial 3D T2-weighted MRI images from the region of the inner ear of (A) a KS patient with normal semicircular canals (asterisk), and (B) a KS patient with hypoplastic semicircular canals (arrow), and an unspecified atrophic area (arrow head) in his retina (C)**.

All 18 probands without an identified molecular genetic cause underlying KS were men. A subset of 7 probands had a history of micropenis, and 4 of them also displayed cryptorchidism, consistent with deficient hypothalamic-pituitary-gonadal (HPG) axis activation in fetal life and/or during infancy. Pubertal development was absent in 15 probands, whereas 3 men had displayed partial puberty. In addition, three probands had CHARGE syndrome-associated features. One of them had hypoplastic lateral and superior left semicircular canals, as well as absent right posterior canal (Figure 3). His child had unilateral microphthalmia and bilateral coloboma. The second proband displayed cleft lip and palate, unilateral microphthalmia and coloboma, bilateral hearing impairment, left facial nerve palsy, cup-shaped ears, upper body muscular atrophy, and hypoplastic semicircular canals. Thus, he fulfilled the diagnostic criteria for CHARGE syndrome [34]. The third proband also had cup-shaped ears and upper body muscular atrophy; unfortunately, MRI scan was not available. However, none of these 3 probands had mutations in *CHD7*.

## Discussion

Our results show that the nationwide minimal incidence of KS was approximately 1 in 48 000 in Finland. The incidence in men (1 in 30 000) was considerably lower than reported among male military conscripts in France (1 in 10 000) [35], and higher than in Sardinia (1 in 84 000) [36]. Military conscript screening, however, may not be the optimal method to ascertain the incidence of KS because the sense of smell is not typically asked for, and patients with mild reproductive phenotype may escape detection. On one hand, our retrospective study approach - to identify all diagnosed KS cases throughout Finland born during a defined time period - does not account for those who remain undiagnosed with KS due to partial pubertal development or unrecognized hyposmia. On the other hand, the proportion of patients with milder reproductive phenotype is relatively small, because, in Finland, the onset and progression of puberty both in boys and in girls is verified by a general practitioner at school welfare clinics, and those without signs of puberty at an appropriate age are referred to a pediatrician or pediatric endocrinologist. The observed sex difference in our minimal incidence estimate of KS was unlikely due to referral bias. This four-fold incidence in men as compared to women was consistent with the estimates for single tertiary referral centers [6,8]. The exact reason for this male predominance is currently unknown. Possible explanations include underdiagnosis in female teenagers, or sex-dependent penetrance of the mutations in unknown KS genes due to either differences in embryonic development or in susceptibility of the HPG axis to disturbances in boys.

In addition to sex-specific difference in the incidence of KS, the molecular genetic diagnoses also differed between sexes. Although *FGFR1 *mutations are not the sole cause of KS in women worldwide [11,22,23,37], all 5 female probands herein carried an *FGFR1 *mutation, a proportion significantly higher than in men. A similar sex-specific difference between *FGFR1 *mutation carriers was also observed among a large series of normosmic HH patients [5]. From the clinical point of view, KS was passed on to offspring via assisted reproductive techniques in two families, suggesting that all patients with congenital HH undergoing infertility treatment should be offered genetic counseling, similarly as in other situations in which an inherited condition in the family is suspected or detected [38].

**Figure 4:**
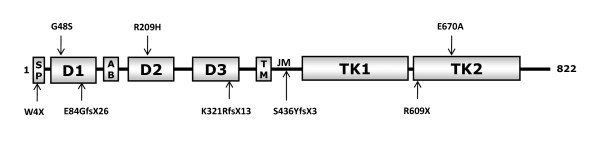
**Schematic of the *FGFR1* mutations at protein level**. SP, signal peptide; D1-D3, immunoglobulin-like domains; TM, transmembrane domain; JM, juxtamembrane domain; TK1-2, tyrosine kinase domain (contains two subdomains). The G48S mutation is located in the first immunoglobulin-like domain (D1), involved in the receptor autoinhibition. The R209H mutation is located in D2, responsible for ligand binding and specificity. The E670A mutation lies within TK2, responsible for activating the MAP (mitogen-activated protein) kinase pathway. The nonsense mutations, W4X and R609X, and the frameshift mutations (E84GfsX26, K321RfsX13, S436YfsX3), all lead to premature stop codons.

All nine identified *FGFR1 *mutations are located in different domains of the receptor (Figure 4), and four of them were novel (W4X, R209H, E670A, and S436YfsX3). The nonsense mutations W4X, and R609X [39], and the frameshift mutations E84GfsX26 [40], K321RfsX13 [40], and S436YfsX3, all lead to premature stop codons. Both missense mutations G48S [20] and E670A, found in patients with familial KS, displayed impaired downstream signaling as assessed by MAPK phosphorylation. G48S is located in the first immunoglobulin-like domain, which is involved in the receptor autoinhibition, and interacts with the second and the third immunoglobulin -like domains. These interactions alter the affinity for ligand and heparin [41,42]. E670A, located in the intracellular tyrosine kinase domain of the receptor, is anticipated to disrupt autophosphorylation of the TK domain [42]. These results were consistent with loss-of-function. On the other hand, R209H displayed relatively normal MAPK signaling, which suggests that another signaling pathway may be impaired by this mutation.

Overall, 40% of the probands, a proportion similar to that observed in other populations [20], could be given a molecular genetic diagnosis. In our series, three (12%) men had *KAL1 *mutations which all are expected to cause loss-of-function of anosmin-1: both the nonsense mutation R262X [43], and the frameshift mutation S158WfsX45, lead to premature stop codons in the region encoding the first fibronectin type III-like repeat of the protein [44], and the deletion of the last nucleotide of exon 8 and the first three nucleotides of intron 8 (g.2357_2360delAgta) abolishes the splice site, and most likely results in an incorrect transcript. All probands with a *KAL1 *mutation had severe congenital HH in combination with synkinesia, and the proband with the R262X mutation also had renal agenesis.

*CHD7 *mutation analysis has been suggested for KS patients with CHARGE syndrome-like features [23]. However, patients with KS or normosmic HH without suggestive features may also carry *CHD7 *mutations [22,45], and even have children with CHARGE syndrome [45]. Thus, *CHD7 *mutation analysis should be considered for all KS patients. None of our three probands with CHARGE syndrome-like features carried mutations in *CHD7*, analyzed both by direct sequencing and MLPA, expanding the phenotypic overlap between KS and CHARGE also in patients without *CHD7 *mutations.

In rare occasions, a congenital HH patient may carry mutation(s) in more than one HH gene; Falardeau et al. [11] showed that mutations in *FGFR1 *and *FGF8 *synergized to cause severe congenital HH in a male patient. Also, mutations in *NELF *have been suggested to modify KS phenotype [10,12], but evidence of its involvement in congenital HH is not thoroughly convincing. In the current work, none of the patients carried presumably pathogenic mutations in more than one gene (*FGFR1 *or *KAL1*). However, two probands with an *FGFR1 *defect did also harbor novel variants in *NELF *(G94S and T505M), but given that the incidence of KS in Finland was 1 in 48 000 and these variants were also present in 6% and 1% of the controls, it is apparent that these variants are not causing congenital HH. Of note, lack of mutations in *PROK2 *and *PROKR2*, now known to cause autosomal recessive KS [13,17,37,46], probably reflects the unique genetic heritage of the Finnish population [47].

To the best of our knowledge, this is the first study where *WDR11 *has been analyzed in a series of KS patients after Kim *et al*. identified this gene by positional cloning of a translocation breakpoint in a KS patient, and discovered heterozygous missense variants in this gene in 6/201 (3%) of patients with KS or normosmic HH [18]. However, we did not detect any mutations, supporting the fact that mutations in *WDR11 *are rarely involved in congenital HH, at least in the Finnish population.

In contrast to women, the majority of male probands remained without identified mutations, implying the existence of still undescribed gene(s) underlying KS in Finnish men. Indeed, sex-dependent penetrance of the mutations in KS genes yet-to-be discovered could contribute to the higher incidence of KS in men worldwide.

## Conclusions

KS is a rare condition with a nationwide minimal incidence estimate of 1:48000 in Finland. In Finnish men, mutations were detected in only *KAL1 *and *FGFR1*, and all women had loss-of-function mutations in *FGFR1*. Some KS patients without *CHD7 *mutations display CHARGE syndrome-associated phenotypic features, implying that, in addition to *CHD7*, there may be other genes associated with both syndromes.

## Competing interests

The authors declare that they have no competing interests.

## Authors' contributions

EML recruited and phenotyped the patients, performed the statistical analyses, and drafted the manuscript. KV performed most of the molecular genetic analyses, performed the functional analyses, and drafted the manuscript. JT participated in the study design and the molecular genetic analyses, and drafted the manuscript. EE and MT participated in the molecular genetic part of the study. LV planned, provided, and interpreted the MRI analyses. TR carried out the study design, participated in recruiting and phenotyping the patients, and drafted the manuscript. All authors read and approved the final manuscript.
